# Cardiovascular Diseases in the Digital Health Era: A Translational Approach from the Lab to the Clinic

**DOI:** 10.3390/biotech11030023

**Published:** 2022-06-30

**Authors:** Ana María Sánchez de la Nava, Lidia Gómez-Cid, Gonzalo Ricardo Ríos-Muñoz, María Eugenia Fernández-Santos, Ana I. Fernández, Ángel Arenal, Ricardo Sanz-Ruiz, Lilian Grigorian-Shamagian, Felipe Atienza, Francisco Fernández-Avilés

**Affiliations:** 1 Department of Cardiology, Instituto de Investigación Sanitaria Gregorio Marañón (IiSGM), Hospital General Universitario Gregorio Marañón, 28007 Madrid, Spain; anasanchezdelanava@gmail.com (A.M.S.d.l.N.); ligomezc@ing.uc3m.es (L.G.-C.); gonzalo.rios.munoz@secardiologia.es (G.R.R.-M.); mariuge@fibhgm.org (M.E.F.-S.); anaisabel.fernandez@cibercv.es (A.I.F.); arenal@secardiologia.es (Á.A.); rsanzruiz@hotmail.com (R.S.-R.); lgrigorian@cibercv.es (L.G.-S.); felipe.atienza@salud.madrid.org (F.A.); 2 Centro de Investigación Biomédica en Red Enfermedades Cardiovasculares, CIBERCV, 28029 Madrid, Spain; 3 Departamento de Bioingeniería e Ingeniería Aeroespacial, Universidad Carlos III de Madrid, 28911 Madrid, Spain; 4 Facultad de Medicina, Universidad Complutense de Madrid, 28040 Madrid, Spain

**Keywords:** cardiovascular diseases, translational medicine, digital health

## Abstract

Translational science has been introduced as the nexus among the scientific and the clinical field, which allows researchers to provide and demonstrate that the evidence-based research can connect the gaps present between basic and clinical levels. This type of research has played a major role in the field of cardiovascular diseases, where the main objective has been to identify and transfer potential treatments identified at preclinical stages into clinical practice. This transfer has been enhanced by the intromission of digital health solutions into both basic research and clinical scenarios. This review aimed to identify and summarize the most important translational advances in the last years in the cardiovascular field together with the potential challenges that still remain in basic research, clinical scenarios, and regulatory agencies.

## 1. Introduction

Digital Health has disrupted the actual panorama by introducing and establishing technology as one of the most useful and rapidly developing tools in the last decade [1]. These technological advances that are based on computing platforms, connectivity, software, and sensors for health care related uses, give a more holistic view of patient health. Through the availability of new data access ways, patients are gaining more control over their health. 

In this sense, modern medicine is constantly evolving, while incorporating more technologies in the analysis, diagnosis, and treatment decisions. These incorporations include the direct collaboration of clinicians, engineers, and basic computational experts to improve data access, reduce costs, and increase overall efficacy. This synergy will ultimately increase quality and personalization at the medical level [1].

In the last years, the outbreak of Artificial Intelligence (AI) has helped to include prediction algorithms as assessment tools to assist clinicians in their diagnostic decisions. On this matter, the inherent capabilities of AI allow researchers to collect and interpret data relationships in digitalized clinical records that can reveal hidden information for the clinician with an inestimable impact in oncology [2], neurology [3], and cardiology fields [4], among others. These capabilities include the automation of tasks such as processing [1], segmenting images [2], or prognosis prediction [3] and translate into a higher efficiency of the processes by reducing time and costs.

Moreover, cardiovascular diseases (CVDs) are the leading cause of death globally, taking an estimated 17,9 million lives each year, corresponding to 32% of all global deaths [5,6]. CVDs are a group of disorders of the heart and blood vessels and include coronary artery disease, cerebrovascular disease, heart failure, valvular heart disease and other conditions. More than four out of five CVD deaths are due to heart attacks and strokes, and one third of these deaths occur prematurely in people under 70 years of age [7]. 

Cardiology has been one of the medical fields where digital health applications are playing a crucial role, not only with the use of wearable technologies but also in relation to clinical applications. Among others, this field has benefited from the use of wireless ECG recordings, implantable loop recorders, cardiac implantable electronic devices with Bluetooth capability, and virtual or mixed-reality tools at operating rooms [1]. 

In this review, we present the most important translation platforms at different levels that have showed major discoveries in the last decade. 

## 2. A Translational Approach in Cardiovascular Diseases: Chimera or Reality?

### 2.1. The Present Breach among Basic Biomedical Research and Clinical Applications 

**Figure 1:**
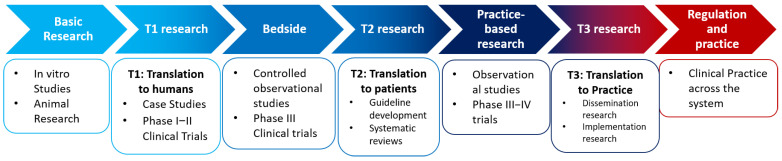
Translational approach from basic research to bedside and clinical practice, describing the three different translational research types.

Translational research aims to transfer the scientific knowledge developed from early research stages into clinical practice across the system. The average time to complete such a transition is 17 years [8], suggesting that efforts need to be made in order to compensate the expensive medical research by improving policy interventions and translation. As a result, intermediate steps have been defined, as described in Figure 1. Based on this, three different translational research types have been identified including (1) the development of treatments and interventions, (2) evaluation of the efficacy and effectiveness of these treatments and interventions and (3) the dissemination and implementation of research for system-wide change [9].

These three types of translational research have been previously described in the literature and highly depend on the stage at which research is being developed [10]. For example, the first one (T1) focuses on translating the basic research findings from preclinical studies, animal research and basic health services research into bedside applications, where controlled observational studies and phase III clinical trials occur.

The second block (T2) includes the translation from bedside to practice-based research, that mainly focuses on phase III and IV trials, observational studies, and survey research. This block is devoted to guideline development, meta-analysis, and systematic reviews with the main objective of translating the information to patients, regulations and practice. 

Finally, the last block (T3) includes the translation from practice-based research to clinical practice across the system, including dissemination and implementation research.

In parallel with this novel classification of the intermediate steps, current legislations have tried to adapt to each of the requirements for protecting patients’ well-being and, at the same time, agility has been introduced into the process [11].

### 2.2. Translational Research as a Highly Complex Structured Matrix

Current trends in preclinical trials include enormous efforts to redesign and evaluate new early phase clinical trial designs [12]. This effort is focused on the identification of biomarkers or endpoints that enable one to identify the full potential and the possible secondary effects of these novel approaches [12,13]. 

Translational research presents a key vision in the development of new drugs and medical devices, although its inclusion in traditional workflows can be both challenging and complex as it involves patients, research, and medical staff in various ways [14].

These approaches are also demanding at the infrastructural level and usually include cutting-edge research, sophisticated machines, complex imaging techniques, and biochemistry laboratories near hospitals and clinics, which are not always available nor possible. 

Additionally, even if the infrastructural level is ensured, the quality of the data and the availability to perform and characterize tests is challenging and can affect the transfer process from the laboratory to the clinic. 

Finally, a clear organized structure where communication is ensured in a multidisciplinary research team is essential for the correct translation of the information. This will ensure good communication among basic scientists and clinicians, will avoid duplication of efforts, and facilitate sharing of key information to identify innovative biomarkers that can be translated into clinical practice.

In this regard, consensus from experts agree that some efforts need to be made including [14]:

- To establish better preclinical models that allow researchers to rationally select target compounds and to better understand their mechanism of action.
- To evaluate and incorporate clear endpoints at preclinical stages that allow for anoptimal evaluation of target-based new drugs.
- To define current monitoring techniques that help to develop the tools, probes, and biological and imaging assays suitable for in vitro assessment, in preclinical models.
- To conduct, in a rapid, coordinated manner, highly specialized, complex, early clinical trials with rigorous standards to deliver complex, detailed data for licensing purposes.
- To ensure a high-quality laboratory infrastructure and expertise with the capacity to provide biological readouts on clinical material in a timely manner.

### 2.3. Current Accomplishments in Cardiovascular Health 

As previously described, CVDs have highly benefited from translational approaches that have already been discussed by several organizations and groups such as the Transnational Alliances for Regenerative Therapies in Cardiovascular Syndromes (TACTICS) [15] or the European Society of Cardiology (ESC) groups, including Digital Health applications for data acquisition and analysis [16]. Some of the most important applications have been summarized in this section.

#### 2.3.1. Translational Bioinformatics 

Translational bioinformatics (TBI) is a well-established field in the study of health informatics that has developed multiple branches of applications such as molecular bioinformatics, biostatistics, statistical genetics, and clinical informatics [17]. The main objective of this approach is to apply informatics to increase the acquisition and analysis of biomedical data, with an emphasis on omics (genomics, metagenomics, epigenomics, transcriptomics, proteomics, metabolomics, phenomics, exposomics, and microbiomics), therefore generating knowledge and medical tools that can be used by both scientists and clinicians with several purposes. Its endpoint explores the improvement of human health by using computer-based information systems, including data mining techniques, to identify patterns or biomarkers that can be used for prediction purposes, as the use of bioinformatics allows us to better understand the molecular basis of cardiovascular diseases and to identify the genes, molecules, and molecular pathways involved. This is not only useful in the identification of potential targets and testing new therapies, but also to predict patient risk, outcome, and the most suitable treatments. All this clinical knowledge is later translated into new application workflows that identify patient clusters, interpreting biological information for treatment selection and health outcome prediction [4].

This revolution, specially at the genomic level, has been possible by the development of next-generation sequencing (NGS) methods that apply different approaches to achieve high-throughput sequencing. These techniques include DNA-seq techniques, such as long-read and short-read sequencing methods, Chromatin Interaction Analysis by Paired-end Tag Sequencing (ChIA-PET), Chromatin Conformation Capture with Sequencing (Hi-C), Assay for Transposase-Accessible Chromatin with High-throughput Sequencing (ATAC-Seq) [5], Chromatin Immunoprecipitation Sequencing (ChIP-seq), gene arrays, and RNA-seq techniques [6]. At the proteomic level, the use of two-dimensional polyacrylamide gel electrophoresis (2DGE), mass spectrometry, and protein arrays allows the massive exploration of protein differences associated with pathological situations.

The impact of this approach in the CVD field is extensive, as most heart diseases are related to a certain genetic component that has highly benefited from the democratization of data and the rise of knowledge of public databases [18]. In this field, both academic, governmental, and industrial initiatives have developed ways to share information at both national and international levels. One of the most important initiatives that has been recently developed is the partnership between the American Heart Association (AHA) Institute for Precision Cardiovascular Medicine and Amazon Web Services by providing a variety of grant funding opportunities for testing and refining AI and machine learning algorithms using healthcare system data, with an aim of promoting precision medicine [19]. 

From the academic perspective, several institutions, including the National Center of Biotechnolgy Information (NCBI), have contributed to the development of portals, analytics platforms, databases, and centralized repositories [7] focusing on cardiovascular diseases. This includes the Knowledge Portal Framework focused on cardiovascular disease, in which HeartBioPortal [8] and the Cerebrovascular Disease Knowledge Portal [9] contain useful gene expression data. Regarding analytic platforms for the development of precision medicine, one could highlight the one from the American Heart Association [10] and from DataSTAGE [7]. Examples of developed databases and central repositories at the genetic level include the Heart Gene Database (HGDB) [11] and the Gene Expression Omnibus (GEO) [12], the COPaKB [13], HeartBD2K [14] and ProteomeXchange [15], among others [16], at the proteomic level, and MetabolomeXchange [17] at the metabolomic level. Others such as the CardioGenBase [20], In-Cardiome [21], Cardio/Vascular Disease Database [18], and dbGap [19] combine gene, functional, drug, and multi-omic studies. In this trend, initiatives such as the IMPaCT platform driven by the Instituto de Salud Carlos III aims to combine predictive medicine, data science, and genomic medicine as a transversal approach to develop precision medicine in the Spanish National Healthcare System. 

In addition to academic efforts, government initiatives have also made available nation’s data through large genomic sequencing programs. Among the most relevant ones, we found the NIH’s All of Us Research Program, which includes many cardiovascular disease phenotypes, demographic information, and physical measurements, as well as whole genome sequencing data [22], the 100K Genomes Project in UK [11], and the 100K Wellness Pioneer Project in China. 

Several companies have also contributed to scaling the use of bioinformating tools by implementing different tests or products that are commercialized in a standardized format. One example of these companies is Illumina, a biotechnology company that offers NGS and later tools for analysis. Many other biotech startup companies and non-profit initiatives have also shared that goal and many have finally effectively integrated the workflow of Illumina and Qiagen [7]. 

Other projects have also contributed to this field by developing and nourishing population-wide multi-omics initiatives such as the NHLBI Trans-Omics for Precision Medicine (TOPMed) program, including the integration of whole-genome sequencing (WGS), metabolic profiles, proteomics, and RNA expression patterns, among others, with molecular, imaging, and clinical data for the study of atherosclerosis [18]. Simpler approaches have explored the use of bioinformatics analysis for coronary heart disease, identifying different genes associated with atherosclerosis [20] and coronary heart disease prediction [23,24,25], as well as for myocardial infarction [26,27], for dilated cardiomyopathy [28], for high blood pressure [29,30,31], and for cardiovascular risk [32,33] and cardiomyopathy in general [34]. Important efforts have also been mounted, revealing the role of the transcriptome [35,36,37], the epigenome [38,39], and the metabolome [40] in these cardiovascular diseases. Recent breakthroughs in sequencing combined with better bioinformatics tools have enabled researchers to analyze the composition of the microbiome and how these microbes are involved in CVD disease. In this context, recent initiatives are evaluating the changes on the metagenome conditioned by diet and its impact on atherosclerosis [22]. 

In summary, there is a clear potential of transforming risk prediction, CVD diagnosis, treatment personalization potentials, and the selection of integration and dose. However, the integration of technology into the clinical care workflow is uneven among institutions [11]. Other limitations in this field include the ethical and legal issues that arise due to the massive production and use of personal data from patients and the rapid evolution of the field, which usually leaves behind its adaptation to clinical practice and bioinformatics. 

#### 2.3.2. Computational Models for Personalized Medicine 

In silico trials are based on computer simulations that contain specific information from the patient, enabling the personalization of the models. The term in silico indicates any use of computers in clinical trials, even if limited to the management of clinical information in a database. 

**Figure 2:**
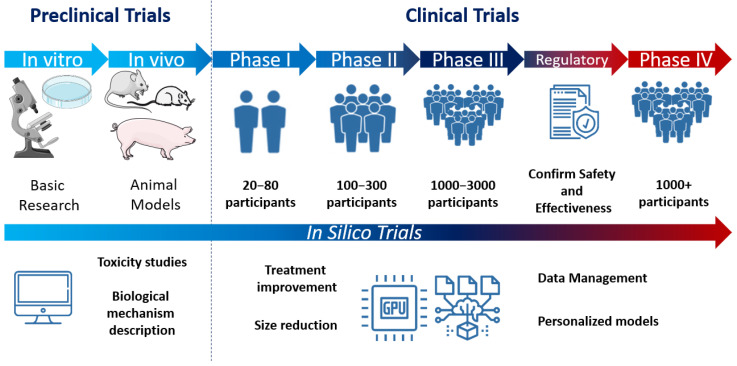
Preclinical and clinical trial development diagram including the type of research used for the characterization of any treatment (basic research, animal models) and the estimated number of participants for each phase in clinical trials.

This type of computation is currently being tested in the development or regulatory evaluation of medicinal products [23,24,25,26], devices, interventions, or in the characterization and modeling of different diseases [27,28,29,30]. Although this approach presents major limitations that will be later commented on [31], the combination of the information extracted from the simulations with clinical information can increase the understanding of biological mechanisms [32,33] (Figure 2). Nowadays, these types of trials are currently being validated at in vitro and in vivo levels, as they are expected to have major benefits over current animal trials.

In silico trials soften these biases by using accurate computer models for a specific treatment and its development, including patient characteristics to broaden the testing scenario to different patient groups and more information. In this sense, the idea of in silico trials is to create a virtual twin in the computer that can test all possible treatments, enabling observation through a computer simulation of how well the candidate biomedical product performs and whether it produces the intended effect without inducing adverse effects. Regarding CVDs, the methodology to obtain the data can vary, from macro anatomical 3D models of a patient obtained from computed tomography or magnetic resonance [41], where electro-mechanical [42] and hemodynamics models can be implemented to mimic the movement and the conduction systems of the heart, to cell-based differential equations emulating every known ionic channel that may affect or modify cardiac cells’ functioning [43,44]. In this line, artificial intelligence brings new tools based on neural networks to predict clinical and anatomical features, e.g., the heart shape based on the MRI and clinical data of the patients (height, weight, sex, heart rate, among others) or the implementation of variational autoencoders on the low ejection fraction data of patients to generate an understandable representation [45] of how the AI performs in its core. This is one known drawback of AI: how it operates or the decisions it makes most of the time are hidden or lack direct interpretation due to its high complexity and huge dimensionality of the transformations performed with real and synthetic data.

Therefore, in silico clinical trials could help to apply the 3Rs of fundamentals (i.e., reduce, refine, and partially replace real clinical trials) by:

(1) Reducing the size or studying specific groups at the clinical level that are identified as risk groups at in silico level.
(2) Adding more detailed information obtained from this type of trials to better understand interactions with different groups and long-term effects that clinical trials cannot provide.
(3) Replacing the preclinical phase and preserving the clinical trial for legal requirements.
(4) Improving unsuccessful treatments or products by providing extra information, as this increases innovation, decreases economical costs, and exponentially increases the understanding of biological processes.
(5) Avoiding the use of animal models by directly including clinical data and personalized information from the patients. This significantly decreases the overall costs associated with the development of treatments and has proven to be more effective at predicting the behavior of the drug or treatment in large-scale trials and identifying secondary effects, therefore better screening the treatments that progress to phase III clinical trials.

As previously mentioned, the validation of these types of experiments highly depends on experimental data from both in vitro and in vivo protocols. This information is devoted to nourishing and calibrating the experimental equations that shape in silico models.

At the CVD level, these studies are present at different levels including cellular studies for the pharmacological testing of new compounds [46], evaluation of drug effects at the tissue level in combination with AI [47], and whole-organ simulation for the evaluation of different treatment strategies [3,48].

#### 2.3.3. In Vitro Research and Translational In Vitro Diagnostics

In vitro research has always represented the first step for the development of drug discovery in preclinical models. Although these assays are essential for the development of molecules, 95% of early-phase studies are eliminated in further stages [34]. The main causes of elimination include deficient properties of the product (45%), lack of efficacy (28%), in vivo toxicity (11%), adverse effects (10%), or commercial purposes (6%).

In the CVD field, the major advance registered in the last decade has been the development and use of in vitro models using induced Pluripotent Stem Cells (iPSC) that can be later differentiated into multiple cardiac cellular types such as cardiomyocytes, cardiac fibroblasts, smooth muscle cells, and endothelial cells [35].

Due to the immature profile of these cells, and in the scope of a translational scenario, multiple strategies have been developed to mimic the properties of native tissue including extracellular matrix hydrogels [36,37], differentiation in 3D structures [38], prolonged culture times [39], hormone addition [40], substrate stiffness [41], biophysical stimulation [42], and in vivo maturation [43].

In vitro diagnostic tests are medical devices that consist of a reagent, calibrator, control material, kit of instruments and materials, apparatus, and equipment or system, used alone or in association with others [34]. They are intended by the manufacturer to be used in vitro for the study of samples from the human body, including blood and tissues. These models have been already explored at the cardiovascular level, usually in combination with digital health tools that enable electronic analysis or data acquisition and further analysis using machine learning [44]. These approaches are usually intended to characterize a physiological or pathological condition [45], to identify a possible congenital anomaly [46], to determine safety and compatibility with potential medical device recipients [47,48], and to monitor therapeutic measurements [49].

Although multiple applications have been described in this field, there are several present drawbacks that limit their generalized use and that are highly conditioned by both the type of samples and the development of the protocols, including:

(1) Inappropriate patient sample or signal acquisition that leads to an inability to analyze the data.
(2) Difficulties or deterioration of the sample during its collection, management, treatment, storage, or transport, especially for biological samples.
(3) Inability to afford in vitro testing at large scales or highly efficient computational systems that can analyze large amounts of data.

In addition, in vitro diagnostic companies are key in this scenario, by taking an active role in collaborating with laboratory professionals, adapting and disseminating evidence-based recommendations about bio-specimen collection into the research settings from preclinical to phase III studies.

#### 2.3.4. Animal Models as a Translational Model for Research

Animal models represent the intermediate transfer point among in vitro cultures and clinical trials. These models are essential for the translation of drug findings from bench to bedside and their critical evaluation regarding their predictive validity is of major importance [50]. For this reason, current trends encourage researchers not only to analyze the results from the lab to the clinic, but also to evaluate the efficacy and efficiency in both directions, identifying clinical bedside findings that were not predicted by animal testing [51].

Furthermore, a proper design, execution, and reporting of animal models is essential to evaluate preclinical data and ensure both reproducibility and translation to the clinic [52,53].

Finally, regulatory agencies play a key role in preclinical testing in animal models as they appear to be an unquestionable data source on the performance of the drug or product.

At the CVD level, animal models can be categorized in two groups: small mammalian animal models of heart disease and large animal models. The most common small animal models include mouse, rat and rabbit animal models with various applications such as myocardial infarction [54,55,56], cryoinjury models [57], hypertensive animals [58], and cardiac electrophysiology models [59]. Large animal models include dogs [60], pigs [61] and goats [62] for a number of different applications in preclinical stages such as drug-induced arrhythmia studies, heart failure, or myocardial ischemia [62].

These models present some disadvantages such as the limited translation of biological products into the clinical scenario and differences between the preclinical models and the target population of patients [63,64]. First, there are significant differences in the cardiac regenerative capacities of rodents and humans, so results obtained in preclinical models may not necessarily translate to humans, especially if the products used as therapeutic products are of murine origin [15,65]. Secondly, animals included in preclinical studies are usually young, and in some cases of only one gender. Preclinical studies should resemble as much as possible the target population of patients, where patients are usually aged, have other comorbidities, and have concomitant routine medications [15,65,66,67].

#### 2.3.5. Signal Acquisition and Processing Automation Using Artificial Intelligence

Recently, AI has presented a major impact in the medical sciences [68] by automatizing tasks and predicting outcomes with unprecedented performance in real-time applications [69,70,71]. These advances are occurring at a fast pace in research laboratories that implement algorithms that need to learn or to be trained to achieve high accuracy performance. The process of training these algorithms implies the use of high-quality data with enough number of samples for the algorithm to learn how to predict. Usually, the more complex the task is, the more data the algorithm will need for high accuracy performance. This trend has already been previously described [49], for example, by comparing biomarker prediction and automatic segmentation. Biomarker prediction, usually implemented by regression analysis, will require a significantly lower number of samples when compared with more complex tasks such as automatic segmentation, implemented by neural networks. As the amount of data needed for this training process presents an exponential tendency, current approaches consider the use of synthetic data from in silico simulations or data produced in the lab to increase the number of samples used for training. Another approach to train these algorithms relies on transfer learning, a process by which a pretrained algorithm from previous experiments is used to calibrate a new one, significantly reducing the number of samples needed for the final process [50]. This approach has been already implemented in the cardiovascular field, showing great performance including complex algorithms such as neural networks [51,52].

Similarly, for other new technologies that have been translated from initial research to widespread clinical practice, it is important to recognize that there will be novel challenges for the clinical deployment of AI tools. Understanding the nature of these new challenges, potential mitigation strategies, and a well-conceived research road map that ensures that advances in AI algorithm development are efficiently translated to clinical practice are of paramount importance [72]. Much of the work in AI is being done at single institutions with single center data for training, testing, and validation of the AI algorithms, lacking the heterogeneity of global data and the effect of population-based factors such as ethnicity, sex, or diet differences among others. A recent review of studies that evaluated the performance of AI algorithms for the diagnostic analysis of medical images found only 6% of the 516 reviewed studies performed external validation [73], and so far, there is limited research demonstrating the generalizability of these algorithms to widespread clinical practice.

In the CVD field, AI has played a major role in the last years [74] by enabling remote data collection [75], the analysis of large populations to identify profiles or groups that better respond to a given treatment [76,77], arrhythmia classification [78,79], and the identification of potential biomarkers for prognostication [80].

### 2.4. Economical Issues and Legal Regulations

Among the most important advantages of combining CVD translational approaches and digital health is the decrease in the average Research and Development (R&D) costs for new medicines, where clinical trials account for nearly 50% of the investment [68].

Regarding legal regulations, data privacy, quality of data and the interpretability of IT systems, as well as intellectual property (IP) rights, they are in the eye of the storm.

At the European level, several regulations are applicable including the European Regulation 2017/745 on medical devices and Regulation (EU) 2017/746 on in vitro diagnostic medical devices (applicable as of 26 May 2022). These are in consonance with the General Data Protection Regulation (EU) 2016/679 (GDPR).

In addition, software that qualifies as a medical device must follow the provisions relating to medical devices, which vary depending on the type and application of a certain medical device. EU Regulation 2017/45 is fully applicable whereas Regulation 2017/746 will remain in a translational situation until 26 May 2022. The European Commission has issued guidelines on the classification of medical devices (MEDDEV Guidelines) and, in particular, on the Qualification and Classification of standalone software used in healthcare. Digital solutions to be adopted by the National Health service are examined to ensure that the required security standards for the public administration are met.

AI in healthcare is mainly regulated by the EU Medical Devices Regulation 2017/745 (MDR) and in vitro Diagnostic Medical Devices Regulation 2017/746 (IVDR) in combination with the GDRP. Medical devices are often either developed using AI or they have an AI component. The GDPR applies since the application of AI implies the collection or treatment of data, and, specifically health data, which is considered as special category data that is subject to strict privacy and data protection obligations.

Moreover, the Ethics Guidelines for Trustworthy AI, published by the European Commission (2019) [81], highlighted that AI applications should not only be consistent with the law, but they must also adhere to ethical principles and ensure their implementations to avoid unintended harm.

Despite all the efforts that have been made to rapidly adapt to a constantly changing scenario, there are some key areas of enforcement for digital health that still need to be addressed:

(1) Regulatory authorities’ actions against digital health and healthcare IT that meet the definition of medical devices but have not obtained the CE mark.
(2) The European Data Protection Agency’s actions in the event of breaches of data protection legislation and data security.

## 3. Current Trends and Future Perspectives

**Figure 3:**
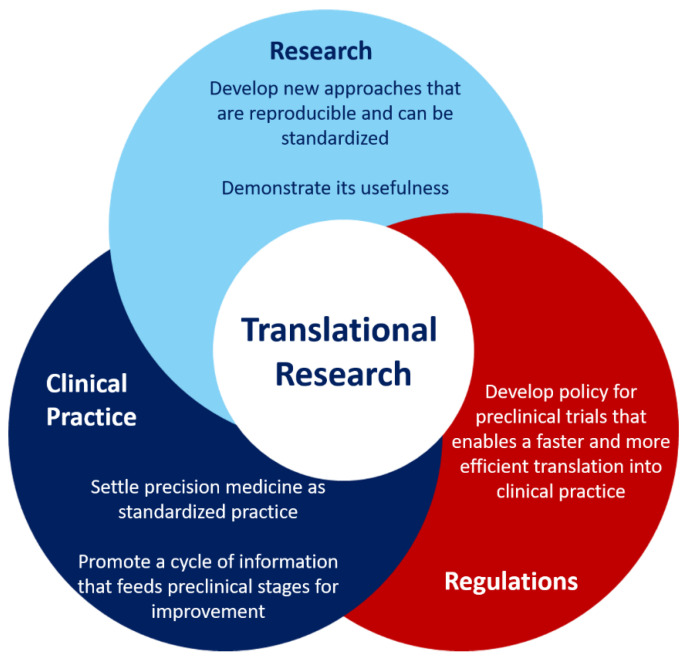
Current challenges and opportunities in the field of translational research.

Although translational research has experienced massive advances in combination with digital health tools, there are some improvements that should be addressed in the upcoming years, summarized in Figure 3.

In particular, standards should be developed for data curation, distribution, sharing, and management to ensure a proper translation from preclinical to clinical scenarios and reproducibility of the results [82] (Figure 3). The most important factors affecting low reproducibility include a lack of access to raw data, misidentified or cross-contaminated samples, inability to properly manage complex datasets, low-quality research practices and experimental design and a competitive culture that rewards novel findings and undervalues negative results [83].

The action needed to overcome these challenges has been already started including different potential improvements in different scenarios. Investigators, institutions, and journals are now demanding the application of good scientific methods and data accessibility from early stages of the research workflow [83].

In addition, policies have to face and overcome their own *valleys of deaths* that are both mainly present in T1 and T2 translational phases. Improvements to ameliorate regulations in translational science include new legislation and regulations, guidance for professionals, standards, and evidence-based guidelines and commercialization and innovation strategies [84].

Finally, at the clinical level, it is necessary to clarify and redesign the concept of evidence-based healthcare to facilitate understanding, analysis, improvement and/or replacement of the process as it is currently conceived, purported, and practiced [85]. Among the most important translational science priorities, some important factors were found, such as the predictive efficacy of preclinical trials, new therapeutic modalities to reach currently inaccessible diseases or pathologies, new methodologies to increase efficiency in preclinical development, identification of new biomarkers for human clinical response prediction, and clinical trial redesigns to facilitate fast clinical practice incorporation [13].

## 4. Conclusions

Digital Health has highly disrupted the cardiovascular panorama by including tools that help to identify biomarkers that can be transferred from preclinical stages into clinical scenarios. In this sense, several advances have been made in bioinformatics, in vitro research, and preclinical animal models to identify and standardize potential biomarkers that can contribute to a successful translation of the results from these scenarios to clinical practice.

This translation has been highly enhanced by the development of new analytical tools that include AI algorithms, by processing and extracting patterns in data from preclinical scenarios to clinical practice. However, a lot of efforts have to be made to continue with this transition and validate the standardization protocols proposed.

This evolution is expected to continue in the upcoming years, leading to the development of new personalized treatments.

## References

[1] Saner H., Van Der Velde E. (2016). eHealth in Cardiovascular Medicine: A Clinical Update. Eur. J. Prev. Cardiol..

[2] Luchini C., Pea A., Scarpa A. (2021). Artificial Intelligence in Oncology: Current Applications and Future Perspectives. Br. J. Cancer.

[3] Pedersen M., Verspoor K., Jenkinson M., Law M., Abbott D.F., Jackson G.D. (2020). Artificial Intelligence for Clinical Decision Support in Neurology. Brain Commun..

[4] De Marvao A., Dawes T.J.W., Howard J.P., O’Regan D.P. (2020). Artificial Intelligence and the Cardiologist: What You Need to Know for 2020. Heart.

[5] Visseren F.L.J., MacH F., Smulders Y.M., Carballo D., Koskinas K.C., Bäck M., Benetos A., Biffi A., Boavida J.M., Capodanno D. (2021). 2021 ESC Guidelines on Cardiovascular Disease Prevention in Clinical Practice. Eur. Heart J..

[6] WHO|Cardiovascular Diseases (CVDs) WHO 2016. https://www.who.int/news-room/fact-sheets/detail/cardiovascular-diseases-(cvds).

[7] Cardiovascular Diseases—World Health Organization. https://www.who.int/health-topics/cardiovascular-diseases#tab=tab_3.

[8] Morris Z.S., Wooding S., Grant J. (2011). The Answer Is 17 Years, What Is the Question: Understanding Time Lags in Translational Research. J. R. Soc. Med..

[9] Westfall J.M., Mold J., Fagnan L. (2007). Practice-Based Research—“Blue Highways” on the NIH Roadmap. JAMA.

[10] Rubio D.M.G., Schoenbaum E.E., Lee L.S., Schteingart D.E., Marantz P.R., Anderson K.E., Platt L.D., Baez A., Esposito K. (2010). Defining Translational Research: Implications for Training. Acad. Med..

[11] Wolf S.M., Clayton E.W., Lawrenz F. (2020). Introduction: The Crucial Role of Law in Supporting Successful Translation of Genomics into Clinical Care. J. Law Med. Ethics.

[12] McMahon G.T., Katz J.T., Thorndike M.E., Levy B.D., Loscalzo J. (2010). Evaluation of a Redesign Initiative in an Internal-Medicine Residency. N. Engl. J. Med..

[13] Austin C.P. (2021). Opportunities and Challenges in Translational Science. Clin. Transl. Sci..

[14] Lehmann F., Lacombe D., Therasse P., Eggermont A.M.M. (2003). Integration of Translational Research in the European Organization for Research and Treatment of Cancer Research (EORTC) Clinical Trial Cooperative Group Mechanisms. J. Transl. Med..

[15] Fernández-Avilés F., Sanz-Ruiz R., Climent A.M., Badimon L., Bolli R., Charron D., Fuster V., Janssens S., Kastrup J., Kim H.S. (2017). Global Position Paper on Cardiovascular Regenerative Medicine. Eur. Heart J..

[16] Svennberg E., Tjong F., Goette A., Akoum N., Di Biaise L., Bordachar P., Boriani G., Burri H., Conte G., Deharo J.-C. (2022). How to Use Digital Devices to Detect and Manage Arrhythmias: An EHRA Practical Guide. EP Eur..

[17] Qazi S., Raza K. (2021). Translational Bioinformatics in Healthcare: Past, Present, and Future. Transl. Bioinform. Healthc. Med..

[18] Khomtchouk B.B., Tran D.T., Vand K.A., Might M., Gozani O., Assimes T.L. (2020). Cardioinformatics: The Nexus of Bioinformatics and Precision Cardiology. Brief. Bioinform..

[19] Houser S.R. (2016). The American Heart Association’s New Institute for Precision Cardiovascular Medicine. Circulation.

[20] Mao C., Howard T.D., Sullivan D., Fu Z., Yu G., Parker S.J., Will R., Vander Heide R.S., Wang Y., Hixson J. (2017). Bioinformatic Analysis of Coronary Disease Associated SNPs and Genesto Identify Proteins Potentially Involved in the Pathogenesis of atherosclerosis. J. Proteom. Genom. Res..

[21] O’Leary K. (2021). AI Refines Treatment Selection for Heart Failure. Nat. Med..

[22] Fernández A.I., Bermejo J., Yotti R., Martínez-Gonzalez M.Á., Mira A., Gophna U., Karlsson R., Al-Daccak R., Martín-Demiguel I., Gutiérrez-Ibanes E. (2021). The Impact of Mediterranean Diet on Coronary Plaque Vulnerability, Microvascular Function, Inflammation and Microbiome after an Acute Coronary Syndrome: Study Protocol for the MEDIMACS Randomized, Controlled, Mechanistic Clinical Trial. Trials.

[23] Li Y., Meng H., Liu Y., Lee B.P. (2015). Fibrin Gel as an Injectable Biodegradable Scaffold and Cell Carrier for Tissue Engineering. Sci. World J..

[24] Passini E., Britton O.J., Lu H.R., Rohrbacher J., Hermans A.N., Gallacher D.J., Greig R.J.H., Bueno-Orovio A., Rodriguez B. (2017). Human In Silico Drug Trials Demonstrate Higher Accuracy than Animal Models in Predicting Clinical Pro-Arrhythmic Cardiotoxicity. Front. Physiol..

[25] Patel D., Stohlman J., Dang Q., Strauss D.G., Blinova K. (2019). Assessment of Proarrhythmic Potential of Drugs in Optogenetically Paced Induced Pluripotent Stem Cell-Derived Cardiomyocytes. Toxicol. Sci..

[26] Vicente J., Zusterzeel R., Johannesen L., Ochoa-Jimenez R., Mason J.W., Sanabria C., Kemp S., Sager P.T., Patel V., Matta M.K. (2019). Assessment of Multi-Ion Channel Block in a Phase I Randomized Study Design: Results of the CiPA Phase I ECG Biomarker Validation Study. Clin. Pharmacol. Ther..

[27] Liberos A., Bueno-Orovio A., Rodrigo M., Ravens U., Hernandez-Romero I., Fernandez-Aviles F., Guillem M.S., Rodriguez B., Climent A.M. (2016). Balance between Sodium and Calcium Currents Underlying Chronic Atrial Fibrillation Termination: An in Silico Intersubject Variability Study. Hear. Rhythm.

[28] Vigmond E., Vadakkumpadan F., Gurev V., Arevalo H., Deo M., Plank G., Trayanova N. (2009). Towards Predictive Modelling of the Electrophysiology of the Heart. Exp. Physiol..

[29] Arevalo H.J., Vadakkumpadan F., Guallar E., Jebb A., Malamas P., Wu K.C., Trayanova N.A. (2016). Arrhythmia Risk Stratification of Patients after Myocardial Infarction Using Personalized Heart Models. Nat. Commun..

[30] Rivera-Juárez A., Hernández-Romero I., Puertas C., Zhang-Wang S., Sánchez-Álamo B., Martins R., Figuera C., Guillem M.S., Climent A.M., Fernández-Avilés F. (2019). Clinical Characteristics and Electrophysiological Mechanisms Underlying Brugada ECG in Patients With Severe Hyperkalemia. J. Am. Hear. Assoc. Cardiovasc. Cerebrovasc. Dis..

[31] Carro J., Rodríguez-Matas J.F., Monasterio V., Pueyo E. (2017). Limitations in Electrophysiological Model Development and Validation Caused by Differences between Simulations and Experimental Protocols. Prog. Biophys. Mol. Biol..

[32] Crumb W.J., Vicente J., Johannesen L., Strauss D.G. (2016). An Evaluation of 30 Clinical Drugs against the Comprehensive in Vitro Proarrhythmia Assay (CiPA) Proposed Ion Channel Panel. J. Pharmacol. Toxicol. Methods.

[33] de la Nava A.M.S., Mansilla A.G., González-Torrecilla E., Ávila P., Datino T., Bermejo J., Arenal Á., Fernández-Avilés F., Atienza F. (2021). Personalized Evaluation of Atrial Complexity of Patients Undergoing Atrial Fibrillation Ablation: A Clinical Computational Study. Biology.

[34] Lippi G., Simundic A.M., Rodriguez-Manas L., Bossuyt P., Banfi G. (2016). Standardizing in Vitro Diagnostics Tasks in Clinical Trials: A Call for Action. Ann. Transl. Med..

[35] Montero P., Flandes-Iparraguirre M., Musquiz S., Pérez Araluce M., Plano D., Sanmartín C., Orive G., Gavira J.J., Prosper F., Mazo M.M. (2020). Cells, Materials, and Fabrication Processes for Cardiac Tissue Engineering. Front. Bioeng. Biotechnol..

[36] Gómez-Cid L., López-Donaire M.L., Velasco D., Marín V., González M.I., Salinas B., Cussó L., García Á., Bravo S.B., Fernández-Santos M.E. (2021). Cardiac Extracellular Matrix Hydrogel Enriched with Polyethylene Glycol Presents Improved Gelation Time and Increased On-Target Site Retention of Extracellular Vesicles. Int. J. Mol. Sci..

[37] Sánchez P.L., Fernández-Santos M.E., Costanza S., Climent A.M., Moscoso I., Gonzalez-Nicolas M.A., Sanz-Ruiz R., Rodríguez H., Kren S.M., Garrido G. (2015). Acellular Human Heart Matrix: A Critical Step toward Whole Heart Grafts. Biomaterials.

[38] Ackermann M., Haake K., Kempf H., Kaschutnig P., Weiss A.C., Nguyen A.H.H., Abeln M., Merkert S., Kühnel M.P., Hartmann D. (2021). A 3D IPSC-Differentiation Model Identifies Interleukin-3 as a Regulator of Early Human Hematopoietic Specification. Haematologica.

[39] Lewandowski J., Rozwadowska N., Kolanowski T.J., Malcher A., Zimna A., Rugowska A., Fiedorowicz K., Łabędź W., Kubaszewski Ł., Chojnacka K. (2018). The Impact of in Vitro Cell Culture Duration on the Maturation of Human Derived from Induced Pluripotent Stem Cells of Myogenic. Cell Transplant..

[40] Yang X., Rodriguez M.L., Leonard A., Sun L., Fischer K.A., Wang Y., Ritterhoff J., Zhao L., Kolwicz S.C., Pabon L. (2019). Fatty Acids Enhance the Maturation of Cardiomyocytes Derived from Human Pluripotent Stem Cells. Stem Cell Rep..

[41] Vuorenpää H., Penttinen K., Heinonen T., Pekkanen-Mattila M., Sarkanen J.R., Ylikomi T., Aalto-Setälä K. (2017). Maturation of Human Pluripotent Stem Cell Derived Cardiomyocytes Is Improved in Cardiovascular Construct. Cytotechnology.

[42] Kroll K., Chabria M., Wang K., Häusermann F., Schuler F., Polonchuk L. (2017). Electro-Mechanical Conditioning of Human IPSC-Derived Cardiomyocytes for Translational Research. Prog. Biophys. Mol. Biol..

[43] Cho G.S., Lee D.I., Tampakakis E., Murphy S., Andersen P., Uosaki H., Chelko S., Chakir K., Hong I., Seo K. (2017). Neonatal Transplantation Confers Maturation of PSC-Derived Cardiomyocytes Conducive to Modeling Cardiomyopathy. Cell Rep..

[44] Saini S.K., Gupta R. (2021). Artificial Intelligence Methods for Analysis of Electrocardiogram Signals for Cardiac Abnormalities: State-of-the-Art and Future Challenges. Artif. Intell. Rev..

[45] Grankvist R., Chireh A., Sandell M., Mukarram A.K., Jaff N., Berggren I., Persson H., Linde C., Arnberg F., Lundberg J. (2020). Myocardial Micro-Biopsy Procedure for Molecular Characterization with Increased Precision and Reduced Trauma. Sci. Rep..

[46] Scholar R., Singaraju J. (2011). Decision Support System for Congenital Heart Disease Diagnosis Based on Signs and Symptoms Using Neural Networks Vanisree K. Int. J. Comput. Appl..

[47] Moore R.A., Madueme P.C., Lorts A., Morales D.L.S., Taylor M.D. (2014). Virtual Implantation Evaluation of the Total Artificial Heart and Compatibility: Beyond Standard Fit Criteria. J. Hear. Lung Transpl..

[48] Abudan A.A., Isath A., Ryan J.D., Henrich M.J., Dugan J.L., Attia Z.I., Ladewig D.J., Dillon J.J., Friedman P.A. (2019). Safety and Compatibility of Smart Device Heart Rhythm Monitoring in Patients with Cardiovascular Implantable Electronic Devices. J. Cardiovasc. Electrophysiol..

[49] Vargas J.E. Home-Based Monitoring of Cardiac Patients. Proceedings of the 1998 IEEE International Conference on Information Technology Applications in Biomedicine, ITAB 1998.

[50] Denayer T., Stöhrn T., Van Roy M. (2014). Animal Models in Translational Medicine: Validation and Prediction. New Horizons Transl. Med..

[51] Van Norman G.A. (2019). Limitations of Animal Studies for Predicting Toxicity in Clinical Trials: Is It Time to Rethink Our Current Approach?. JACC Basic Transl. Sci..

[52] Lawrence C.L., Bridgland-Taylor M.H., Pollard C.E., Hammond T.G., Valentin J.-P. (2006). A Rabbit Langendorff Heart Proarrhythmia Model: Predictive Value for Clinical Identification of Torsades de Pointes. Br. J. Pharmacol..

[53] von Kortzfleisch V.T., Karp N.A., Palme R., Kaiser S., Sachser N., Richter S.H. (2020). Improving Reproducibility in Animal Research by Splitting the Study Population into Several ‘Mini-Experiments’. Sci. Rep..

[54] Wu Y., Yin X., Wijaya C., Huang M.H., McConnell B.K. (2011). Acute Myocardial Infarction in Rats. J. Vis. Exp..

[55] Lindsey M.L., Brunt K.R., Kirk J.A., Kleinbongard P., Calvert J.W., de Castro Brás L.E., DeLeon-Pennell K.Y., Del Re D.P., Frangogiannis N.G., Frantz S. (2021). Guidelines for in Vivo Mouse Models of Myocardial Infarction. Am. J. Physiol. Hear. Circ. Physiol..

[56] Fujita M., Morimoto Y., Ishihara M., Shimizu M., Takase B., Maehara T., Kikuchi M. (2004). A New Rabbit Model of Myocardial Infarction without Endotracheal Intubation. J. Surg. Res..

[57] Polizzotti B.D., Ganapathy B., Haubner B.J., Penninger J.M., Kühn B. (2016). A Cryoinjury Model in Neonatal Mice for Cardiac Translational and Regeneration Research. Nat. Protoc..

[58] Wang W., Liu R., Cao G., Zhang F., Zhang Y., Zhang Z., Wu S. (2010). A Reliable Rabbit Model for Hyperkinetic Pulmonary Hypertension. J. Thorac. Cardiovasc. Surg..

[59] Odening K.E., Baczko I., Brunner M., Mechanisms K.G., de Medeiros R.A., Hausen Z.A. (2020). Animals in Cardiovascular Research: Important Role of Rabbit Models in Cardiac Electrophysiology. Eur. Heart J..

[60] Powers J.C., Recchia F. (2018). Canine Model of Pacing-Induced Heart Failure. Methods Mol. Biol..

[61] Crisóstomo V., Sun F., Maynar M., Báez-Díaz C., Blanco V., Garcia-Lindo M., Usón-Gargallo J., Sánchez-Margallo F.M. (2016). Common Swine Models of Cardiovascular Disease for Research and Training. Lab Anim..

[62] Tsang H.G., Rashdan N.A., Whitelaw C.B.A., Corcoran B.M., Summers K.M., MacRae V.E. (2016). Large Animal Models of Cardiovascular Disease. Cell Biochem. Funct..

[63] Chamuleau S.A.J., Van Der Naald M., Climent A.M., Kraaijeveld A.O., Wever K.E., Duncker D.J., Fernández-Avilés F., Bolli R. (2018). Translational Research in Cardiovascular Repair a Call for a Paradigm Shift. Circ. Res..

[64] Povsic T.J., Sanz-Ruiz R., Climent A.M., Bolli R., Taylor D.A., Gersh B.J., Menasché P., Perin E.C., Pompilio G., Atsma D.E. (2021). Reparative Cell Therapy for the Heart: Critical Internal Appraisal of the Field in Response to Recent Controversies. ESC Hear. Fail..

[65] Grigorian-Shamagian L., Sanz-Ruiz R., Climent A., Badimon L., Barile L., Bolli R., Chamuleau S., Grobbee D.E., Janssens S., Kastrup J. (2021). Insights into Therapeutic Products, Preclinical Research Models, and Clinical Trials in Cardiac Regenerative and Reparative Medicine: Where Are We Now and the Way Ahead. Current Opinion Paper of the ESC Working Group on Cardiovascular Regenerative and Reparative Medicine. Cardiovasc. Res..

[66] Madonna R., Van Laake L.W., Davidson S.M., Engel F.B., Hausenloy D.J., Lecour S., Leor J., Perrino C., Schulz R., Ytrehus K. (2016). Position Paper of the European Society of Cardiology Working Group Cellular Biology of the Heart: Cell-Based Therapies for Myocardial Repair and Regeneration in Ischemic Heart Disease and Heart Failure. Eur. Heart J..

[67] Grigorian Shamagian L., Madonna R., Taylor D., Climent A.M., Prosper F., Bras-Rosario L., Bayes-Genis A., Ferdinandy P., Fernández-Avilés F., Izpisua Belmonte J.C. (2019). Perspectives on Directions and Priorities for Future Preclinical Studies in Regenerative Medicine. Circ. Res..

[68] Allen B., Seltzer S.E., Langlotz C.P., Dreyer K.P., Summers R.M., Petrick N., Marinac-Dabic D., Cruz M., Alkasab T.K., Hanisch R.J. (2019). A Road Map for Translational Research on Artificial Intelligence in Medical Imaging: From the 2018 National Institutes of Health/RSNA/ACR/The Academy Workshop. J. Am. Coll. Radiol..

[69] Sanchez de la Nava A.M., Arenal Á., Fernández-Avilés F., Atienza F. (2021). Artificial Intelligence-Driven Algorithm for Drug Effect Prediction on Atrial Fibrillation: An in Silico Population of Models Approach. Front. Physiol..

[70] Sánchez de la Nava A.M., Atienza F., Bermejo J., Fernández-Avilés F. (2021). Artificial Intelligence for a Personalized Diagnosis and Treatment of Atrial Fibrillation. Am. J. Physiol. Circ. Physiol..

[71] Ríos-Muñoz G.R., Fernández-Avilés F., Arenal Á. (2022). Convolutional Neural Networks for Mechanistic Driver Detection in Atrial Fibrillation. Int. J. Mol. Sci..

[72] Deo R.C. (2020). Machine Learning in Medicine: Will This Time Be Different?. Circulation.

[73] Kim D.W., Jang H.Y., Kim K.W., Shin Y., Park S.H. (2019). Design Characteristics of Studies Reporting the Performance of Artificial Intelligence Algorithms for Diagnostic Analysis of Medical Images: Results from Recently Published Papers. Korean J. Radiol..

[74] Yan Y., Zhang J.W., Zang G.Y., Pu J. (2019). The Primary Use of Artificial Intelligence in Cardiovascular Diseases: What Kind of Potential Role Does Artificial Intelligence Play in Future Medicine?. J. Geriatr. Cardiol..

[75] Cowie M.R., Lam C.S.P. (2021). Remote Monitoring and Digital Health Tools in CVD Management. Nat. Rev. Cardiol..

[76] Sharma A., Zheng Y., Ezekowitz J.A., Westerhout C.M., Udell J.A., Goodman S.G., Armstrong P.W., Buse J.B., Green J.B., Josse R.G. (2022). Cluster Analysis of Cardiovascular Phenotypes in Patients With Type 2 Diabetes and Established Atherosclerotic Cardiovascular Disease: A Potential Approach to Precision Medicine. Diabetes Care.

[77] Jahangiry L., Abbasalizad Farhangi M., Najafi M., Sarbakhsh P. (2021). Clusters of the Risk Markers and the Pattern of Premature Coronary Heart Disease: An Application of the Latent Class Analysis. Front. Cardiovasc. Med..

[78] Shao M., Bin G., Wu S., Bin G., Huang J., Zhou Z. (2018). Detection of Atrial Fibrillation from ECG Recordings Using Decision Tree Ensemble with Multi-Level Features. Physiol. Meas..

[79] Yıldırım Ö., Pławiak P., Tan R.S., Acharya U.R. (2018). Arrhythmia Detection Using Deep Convolutional Neural Network with Long Duration ECG Signals. Comput. Biol. Med..

[80] Krittanawong C., Virk H.U.H., Bangalore S., Wang Z., Johnson K.W., Pinotti R., Zhang H.J., Kaplin S., Narasimhan B., Kitai T. (2020). Machine Learning Prediction in Cardiovascular Diseases: A Meta-Analysis. Sci. Rep..

[81] High-Level Expert Group on Artificial Intelligence Set Up by the European Commission Ethics Guidelines for Trustworthy AI. https://ec.europa.eu/futurium/en/ai-alliance-consultation/guidelines.1.html.

[82] Six Factors Affecting Reproducibility in Life Science Research and How to Handle Them. https://www.nature.com/articles/d42473-019-00004-y.

[83] Begley C.G., Ioannidis J.P.A. (2015). Reproducibility in Science: Improving the Standard for Basic and Preclinical Research. Circ. Res..

[84] Meslin E.M., Blasimme A., Cambon-Thomsen A. (2013). Mapping the Translational Science Policy “Valley of Death”. Clin. Transl. Med..

[85] Pearson A., Jordan Z., Munn Z. (2012). Translational Science and Evidence-Based Healthcare: A Clarification and Reconceptualization of How Knowledge Is Generated and Used in Healthcare. Nurs. Res. Pract..

